# Oxymatrine-associated protection in an MPTP mouse model is accompanied by increased miR-141-3p and reduced HMGB1

**DOI:** 10.3389/fnmol.2026.1731850

**Published:** 2026-02-05

**Authors:** Ping Gan, Qi Zhao, Xing Yi, Yi-Ming Hua, Yu-Fang Ding, Zhi-Mei Huang, Xing Ye, Jian Wu

**Affiliations:** 1 Department of Pharmacy, The Affiliated Taizhou Second People’s Hospital of Yangzhou University, Taizhou, China; 2 Department of Histology and Embryology, Medical School, Nantong University, Nantong, China

**Keywords:** high-mobility group protein B1, microglia, miR-141-3p, neuroinflammation, Oxymatrine, Parkinson’s disease

## Abstract

**Introduction:**

Oxymatrine (OMT) alleviates damage to dopaminergic (DA) neurons and microglia-mediated neuroinflammation in an MPTP mouse model of parkinsonism by inhibiting the High-mobility group protein B1 (HMGB1) pathway. However, the precise mechanism by which OMT inhibits HMGB1 remains unclear. Although miR-141-3p is downregulated in the peripheral blood serum of Parkinson’s disease (PD) patients, its potential relationship with HMGB1 remains unclear.

**Methods:**

TargetScan software and dual-luciferase reporter gene assays predicted that miR-141-3p binds to the 3’-UTR of HMGB1 mRNA. BV2 cells were transfected with miR-141-3p mimics and stimulated with MPP^+^*in vitro* experiments. C57BL/6 J mice received stereotaxic injections of miR-141-3p agomir or miR-141-3p antagomir into the bilateral substantia nigra pars compacta (SNpc). Subsequently, the mice were intraperitoneally injected with MPTP four times within a single day. After miR-141-3p antagomir injection, OMT was administered continuously by injection for 7 days. Behavioral tests were assessed using the rotarod and open field tests. Real-time PCR, western blot, ELISA, and immunofluorescence staining were performed on BV2 cells and SNpc tissues.

**Results:**

Our study showed an inverse correlation between HMGB1 and miR-141-3p expression in both BV2 microglia exposed to MPP^+^ and MPTP-treated mice. TargetScan analysis identified complementary binding sites between miR-141-3p and the 3’-UTR of HMGB1 mRNA, which was subsequently confirmed through dual-luciferase reporter assays. Through experiments in BV2 microglia exposed to MPP^+^ and in MPTP-treated mice, miR-141-3p downregulates HMGB1, reduces pro-inflammatory cytokine readouts, and *in vivo* is associated with improved rotarod and open-field performance and attenuated Tyrosine Hydroxylase (TH)-positive neuronal loss. OMT increases miR-141-3p in the MPTP model, alongside reduced HMGB1 and inflammatory readouts, and these effects are diminished by miR-141-3p inhibition.

**Conclusion:**

miR-141-3p targets HMGB1 to inhibit microglial reaction and mitigate neuroinflammation both *in vivo* and vitro experiments, reduce TH-positive neuronal loss in the MPTP model. OMT increases miR-141-3p in the MPTP model, alongside reduced HMGB1 and inflammatory readouts, and these effects are diminished by miR-141-3p inhibition.

## 1 Introduction

Parkinson’s disease (PD) involves the formation of Lewy bodies containing abundant *α*-synuclein (α-Syn) and the loss of dopaminergic (DA) neurons in the substantia nigra pars compacta (SNpc) (Kalia and Lang, 2015). Reactive microglia are abundantly present in the SNpc of PD patients, and positron emission tomography imaging has demonstrated that microglial reaction is an early and sustained feature of PD (Gerhard et al., 2006). Similarly, PD models—including those induced by lipopolysaccharide (LPS) (Dutta et al., 2008) or genetic modifications (Theodore et al., 2008)—exhibit significant microglial reactivity alongside DA neuron degeneration. Therefore, the reactive microglia is closely related to the progression of PD (Stefanova, 2022). In PD, reactive microglia can release many inflammatory factors, such as IL-1β, IL-12, TNF-*α*, NO, reactive oxygen species, and prostaglandins. These inflammatory factors ultimately lead to the damage or even death of DA neurons, thereby promoting the occurrence and progression of PD (Ho, 2019).

High-mobility group protein B1 (HMGB1) is a highly conserved nuclear protein ubiquitous in mammalian cells. In rats and mice, its protein sequence is identical, but differs from human HMGB1 by two amino acid substitutions within the C-terminal repeat region. HMGB1 comprises three domains: an evolutionarily conserved A-box at the N-terminus, a C-tail at the carboxyl terminus containing 30 repetitive aspartic and glutamic acid residues that modulate DNA binding, and a central B-box that mediates inflammatory responses (Angelopoulou et al., 2018). Studies indicate that HMGB1 can be co-secreted by glial cells and degenerating neurons, where it initiates and amplifies neuroinflammatory immune responses. These HMGB1-driven processes contribute significantly to the pathogenesis of neurodegenerative diseases (Andersson et al., 2018).

MicroRNAs (miRs) are non-coding RNAs consisting of 20–22 nucleotides, which usually regulate the expression of target genes by binding to the 3′-untranslated region (3’-UTR). This interaction occurs through complementary base-pairing, which often highly catalyze the degradation of target mRNAs, thereby regulating the expression level of target gene sequences during the transcriptional process (Ameres and Zamore, 2013). Over 70% of known miRNAs are abundant in the brain, where they play critical roles in neural development (Kosik, 2006), including miR-134, miR-135, miR-let-7 g, miR-101, miR-181a-b, miR-191, miR-124, miR-let-7c, miR-let-7a, miR-29a, and miR-107, etc. (Shao et al., 2010; Adlakha and Saini, 2014). Studies have shown that miR-141-3p is downregulated in the serum of PD patients (Dong et al., 2016; Tolosa et al., 2018). However, its functional role in PD remains unclear.

Oxymatrine (OMT), a natural quinolizidine alkaloid extracted from *Sophora flavescens* Ait, serves as an effective ingredient in traditional Chinese medicine for treating inflammation (Guzman et al., 2013; Zhang et al., 2013; Jiang et al., 2015). It also demonstrates significant efficacy in treating neuroinflammation and providing neuroprotective effects (Mao et al., 2012; Zhang et al., 2013). In 2020, we discovered that OMT can alleviate the damage to DA neurons and the neuroinflammation mediated by microglia in the PD model of mice by inhibiting the HMGB1 pathway (Gan et al., 2020). However, the precise mechanism by which OMT inhibits HMGB1 remains unclear.

In this study, a series of experiments were first conducted to confirm that miR-141-3p can downregulate the HMGB1 expression. Subsequently, an in-depth exploration was carried out to elucidate how miR-141-3p regulates HMGB1 to weaken microglial reaction in MPP^+^-treated BV2 microglia and MPTP-treated mice, reduce the loss of Tyrosine Hydroxylase (TH-positive) neurons in the MPTP model. Additionally, we further discovered that OMT can upregulate miR-141-3p, along with a decrease in HMGB1 expression *in vivo* experiments, inhibits excessive microglial reaction, and ultimately downregulates the release of inflammatory factors from microglia and safeguards neurons.

## 2 Materials and methods

### 2.1 Animals

All experimental animals utilized in this study were male C57BL/6 J mice (23–25 g, 8–10 weeks old), procured from the Laboratory Animal Center of Nantong University. The mice were housed in a specific pathogen-free (SPF) environment under a 12-h light/dark cycle with ad libitum access to food and water. All experimental procedures involving animals were handled in accordance with the Guide for the Care and Use of Laboratory Animals, with experimental protocols approved by the Animal Ethics Committee of Nantong University (approval number: S20240308-009). All experimental procedures followed recommendations in the ARRIVE guidelines.

### 2.2 MPTP-induced acute PD model

Mice were randomly assigned to Control, MPTP, or MPTP + OMT groups. Mice in the MPTP group received intraperitoneal injections of MPTP·HCl (20 mg/kg, M0896, Sigma-Aldrich) four times in a single day at 2-h intervals, following an established protocol for the MPTP model (Jackson-Lewis and Przedborski, 2007). Mice in the MPTP + OMT group were administered OMT (20 mg/kg, PHL89748, Sigma-Aldrich) intraperitoneally 2 h before the first MPTP injection and received another dose of OMT 2 h after the fourth MPTP injection. Starting from day 2, OMT was administered once daily until tissue collection on day 7. Control mice received intraperitoneal injections of an equal volume of 0.9% NaCl.

### 2.3 Stereotaxic injection

Following anesthesia with isoflurane inhalation, mice received intracranial injections into the bilateral SNpc (stereotaxic coordinates relative to Bregma: anteroposterior −3.28 mm, mediolateral ±1.2 mm, dorsoventral −4.7 mm) of either 0.5 nmol miR-141-3p agomir, miR-141-3p agomir negative control (NC agomir), miR-141-3p antagomir, or miR-141-3p antagomir negative control (NC antagomir) (OBiO Technology Shanghai Corp., Ltd.), dissolved in 2.5 μL PBS. A 10-μL Hamilton syringe was used for injection, delivered over 10 min via a syringe pump (Harvard Apparatus). The needle was left in place for an additional 10 min post-injection before slow withdrawal. Mice were kept warm until fully recovered.

### 2.4 Rotarod test

A rotarod apparatus (Harvard Apparatus) was used to assess motor coordination and balance. Prior to formal testing, mice underwent a three-day training period on the rotarod at a constant speed of 5 rpm/min. On the fourth day, an accelerated rotarod test was performed in which the rotation speed increased from 5 rpm/min to 40 rpm/min over a 5- min period. Only mice capable of remaining on the rod for 3–5 min were selected for subsequent experiments. Seven days after MPTP injection, each mouse was tested three times, and the average latency to fall was recorded as a measure of motor function. A maximum latency of 300 s was assigned if a mouse did not fall within the 5-min test period.

### 2.5 Open field test

The open field test was conducted using an arena (40 cm × 40 cm × 40 cm) equipped with an automated data acquisition system (Shanghai Xinruan Information Technology Co. Ltd.). Mice were individually placed in the open arena and allowed to freely explore for 5 min before a 15-min recording session. To get rid of the odor from each previous mouse, the instruments were cleaned with 75% ethyl alcohol. The total distance traveled, average speed, time spent in the center zone, and distance moved in the center zone were collected. Calculated.

### 2.6 Preparation of brain samples

After euthanizing the mice (24–27 g) with excessive inhalation isoflurane, mice were immediately subjected to transcardiac perfusion fixation. Whole brains were carefully extracted and post-fixed in 4% PFA at 4 °C for 12 h, followed by dehydration in 30% sucrose until sedimentation occurred. These samples were reserved for subsequent immunofluorescence staining. Another group of brains was promptly dissected to isolate the ventral midbrain containing SNpc (stereotaxic range: anteroposterior −2.92 to −3.88 mm from Bregma), which were immediately snap-frozen in liquid nitrogen for subsequent analyses including real-time PCR, Western blot, and ELISA.

### 2.7 Immunofluorescence staining

Brain tissue sections (20 μm) were cut on a cryostat (Leica) and floated in PBS for three washes. Sections were then blocked with 3% BSA containing 0.1% Triton X-100 for 1 h, followed by incubation at 4 °C for 24 h with the following primary antibodies: Rabbit anti-AIF-1/Iba1 Antibody (1:1000, 019–19,741, FUJIFILM Wako Pure Chemical Corporation) and Goat anti-Tyrosine Hydroxylase Antibody (1:500, ab317785, abcam). After PBS washes, sections were incubated overnight at 4 °C in the dark with secondary antibodies: Donkey anti-Rabbit IgG Alexa Fluor™ 488 (1:500, A21206, Thermo Scientific) and Donkey anti-Goat IgG Alexa Fluor™ 568 (1:500, A11053, Thermo Scientific). Following another PBS wash, nuclei were stained with Hoechst.

The quantitative statistical analysis used the ratio of the number of Iba1-positive microglia to the area of TH-positive neurons. This analytical approach was adopted because that simply counting the number of IBA1-positive microglia and the area of TH-positive neurons would vary due to different sections. Sections with higher TH-positive neuron density exhibit proportionally greater microglial proliferation following neuronal loss, and vice versa. Therefore, the microglia number-to-DA neuron area ratio provides a more accurate representation of pathological changes. All immunohistochemical analyses were conducted on SNpc-region sections selected from mouse brain atlases, with focus on sections displaying maximal microglial density per sample.

### 2.8 Cell culture and transfection

The microglial BV2 cells were maintained in DMEM (Gibco) supplemented with 10% fetal bovine serum (FBS) and 1% penicillin–streptomycin at 37 °C in a humidified 5% CO₂ incubator. One day prior to transfection, cells were seeded in and transfection was performed when cells reached approximately 60% confluence. BV2 cells were transfected with 100 nM miR-141-3p mimics or miR-141-3p mimics negative control (NC mimics) using LipofectMax transfection reagent for 6 h, followed by replacement with fresh DMEM containing 10% FBS for 48 h. Subsequently, the transfected cells were stimulated with 1 mM MPP^+^ (Sigma-Aldrich) for 24 h.

### 2.9 Dual-luciferase reporter evaluation

Bioinformatic analysis via TargetScan predicted a complementary binding site between miR-141-3p and the 3’-UTR of HMGB1 mRNA. To validate this interaction, the mutant reporter vector pMIR-REPORT Luciferase-HMGB1 3’-UTR (MUT) and wild-type reporter vector pMIR-REPORT Luciferase-HMGB1 3’-UTR (WT) were co-transfected with either miR-141-3p mimics or negative control (NC mimics) (OBiO Technology Shanghai Corp., Ltd.) into U251 cell lines. Luciferase activity was measured using a dual-luciferase reporter assay system 48 h post-transfection.

### 2.10 Real-time PCR

Total RNA from cells and animal tissues was extracted using the Rapid RNA Extraction Kit and TRIzol™ Reagent (Vazyme Biotech Co., Ltd.), respectively, followed by reverse transcription with the HiScript III RT SuperMix for qPCR Kit. Quantitative real-time PCR was performed using a BioRad CFX96TOUCH Real-Time PCR instrument. Gene expression levels normalized to 18S was conducted with the 2^−△△^CT method. The primer sequences are listed in Supplementary Table 1.

### 2.11 Elisa

Levels of HMGB1, tumor necrosis factor-*α* (TNF-α) and interleukin-6 (IL-6) in BV2 cell culture supernatants or the ventral midbrain containing the SNpc were tested by ELISA kits (USCN Life Science Inc). Tissue homogenates were sonicated until clarification, followed by centrifugation at 4 °C for 5 min, with the supernatant aspirated as test samples. A volume of 100 μL of each standard or test sample was added to the wells and incubated at 37 °C for 1 h. After aspiration, 100 μL of detection antibody was added and incubated for 1 h at 37 °C. The plate was then washed, and 100 μL of horseradish peroxidase (HRP)-conjugated secondary antibody was added, followed by incubation for 30 min at 37 °C. After five washes, a substrate solution was added and incubated for 20 min at 37 °C in the dark. The reaction was stopped, and absorbance was measured at 450 nm.

### 2.12 Western blot analysis

The ventral midbrain containing the SNpc or BV2 cell were lysed using RIPA lysis buffer (Beyotime) containing a protease inhibitor cocktail (Roche). Following the protein concentration was determined by BCA assay, equal amounts of protein were separated by SDS-PAGE and transferred to PVDF membranes. After blocking with 5% skim milk for 1 h, the membranes were incubated overnight at 4 °C with the following primary antibodies: β-actin monoclonal antibody (1:8000, A5441, Sigma-Aldrich) and anti-HMGB1 antibody (1:25000, ab79823, abcam). Subsequently, membranes were incubated for 2 h at room temperature with horseradish peroxidase (HRP)-conjugated secondary antibodies: goat anti-mouse IgG HRP (1:8000, A3682, Sigma-Aldrich) and goat anti-rabbit IgG HRP (1:8000, A0545, Sigma-Aldrich). Protein bands were visualized using enhanced chemiluminescence, and images were captured with an automated chemiluminescence imaging system (Tanon). Band intensities were quantified using ImageJ software, and normalized to β-actin levels.

### 2.13 Statistical analysis

All statistical analyses were performed using GraphPad Prism version 9.5.0. Shapiro–Wilk normality test and Levene’s test were used to assess data normality and homogeneity of variance, respectively. Comparisons between two groups were analyzed using Student’s *t*-test. For comparisons among multiple groups, one-way analysis of variance (ANOVA) plus Tukey’s *post hoc* test was employed. Data are presented as mean ± SEM. Statistical significance was set at *p* < 0.05.

## 3 Results

### 3.1 The negative post - transcriptional targeted regulatory effect of miR-141-3p on HMGB1

**Figure 1:**
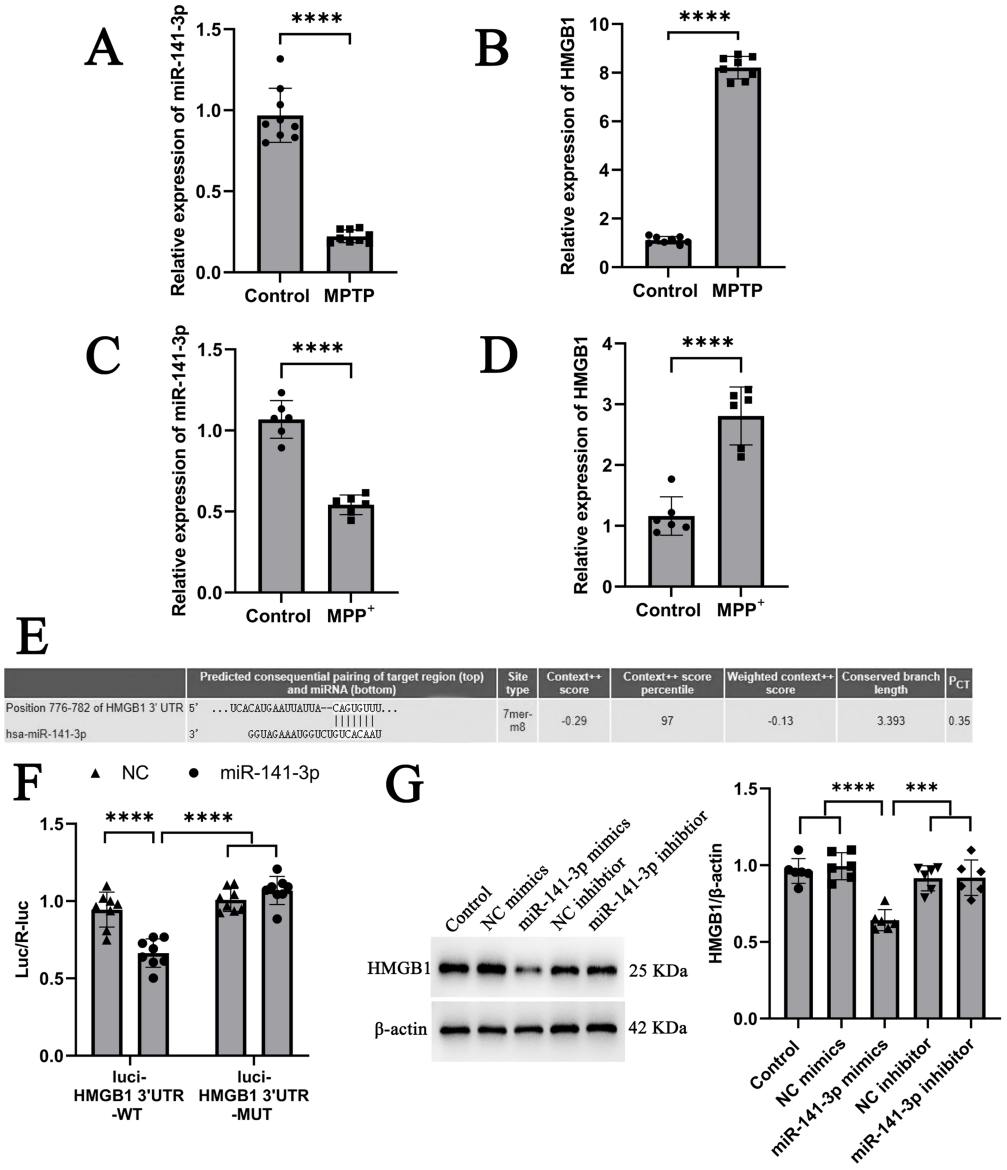
miR-141-3p specifically targeted and regulated HMGB1. **(A,B)** Histogram showed qRT-PCR assay to determine mRNA levels of miR-141-3p (**A**, *n* = 9 per group, ****p < 0.0001, t-test) and HMGB1 (**B**, *n* = 8 per group, ****p < 0.0001, t-test) in the mouse SNpc at 8 d after acute injection of MPTP. (**C,D**) Histogram showed qRT-PCR assay to determine mRNA levels of miR-141-3p **(C)** and HMGB1 **(D)** in BV2 cells at 24 h after MPP^+^ treatment (*n* = 6 per group, ****p < 0.0001, *t*-test). **(E)** miR-141-3p binding sites in HMGB1 3′-UTR predicted by TargetScan database **(F)** Histogram showed dual-luciferase reporter gene activity in U251 cells transfected with wild type (WT) or mutated (MUT) reporter of HMGB1 3′-UTR in the presence of miR-141-3p mimics (miR-141-3p) or negative control (NC) (*n* = 8 per group, ****p < 0.0001, one-way ANOVA). **(G)** Histogram showed western blotting assay to determine HMGB1 expression in U251 cells at 48 h after transfection (*n* = 6 per group, ***p < 0.001, ****p < 0.0001, one-way ANOVA).

A mouse model of PD was established using MPTP. Results revealed a significant downregulation of miR-141-3p mRNA expression in the SNpc region of the mice (Figure 1A), concurrently with a marked upregulation of HMGB1 mRNA expression (Figure 1B). *In vitro* experiments were performed using the mouse microglial cell line BV2. After stimulation with MPP^+^, consistent results were obtained, showing an inverse relationship between miR-141-3p and HMGB1 expression (Figures 1C,D). Bioinformatics analysis using TargetScan software predicted that miR-141-3p binds complementarily to the 3’-UTR of HMGB1 mRNA (Figure 1E). This prediction was further verified by dual-luciferase reporter gene assays. The mutant (MUT) or wild-type (WT) HMGB1 mRNA 3’-UTR reporter gene was cloned into the dual-luciferase reporter vector (pMIR-REPORT). In U251 cells transfected with miR-141-3p mimics, the luciferase activity of the pMIR-REPORT Luciferase-HMGB1 3’-UTR (WT) reporter gene was significantly reduced. In contrast, when the predicted miR-141-3p binding site in the HMGB1 mRNA 3’-UTR was mutated, luciferase activity remained unaltered (Figure 1F). Western blot analysis in U251 cells confirmed that transfection of miR-141-3p mimics led to a decrease in HMGB1 protein expression, while transfection of miR-141-3p inhibitor had no significant effect on HMGB1 protein levels (Figure 1G). The above experimental results demonstrate that miR-141-3p specifically binds to the 3’-UTR of HMGB1 and effectively downregulates its expression.

### 3.2 miR-141-3p reduces the expression of HMGB1 and pro-inflammatory factors in BV2 cells stimulated by MPP^+^

**Figure 2:**
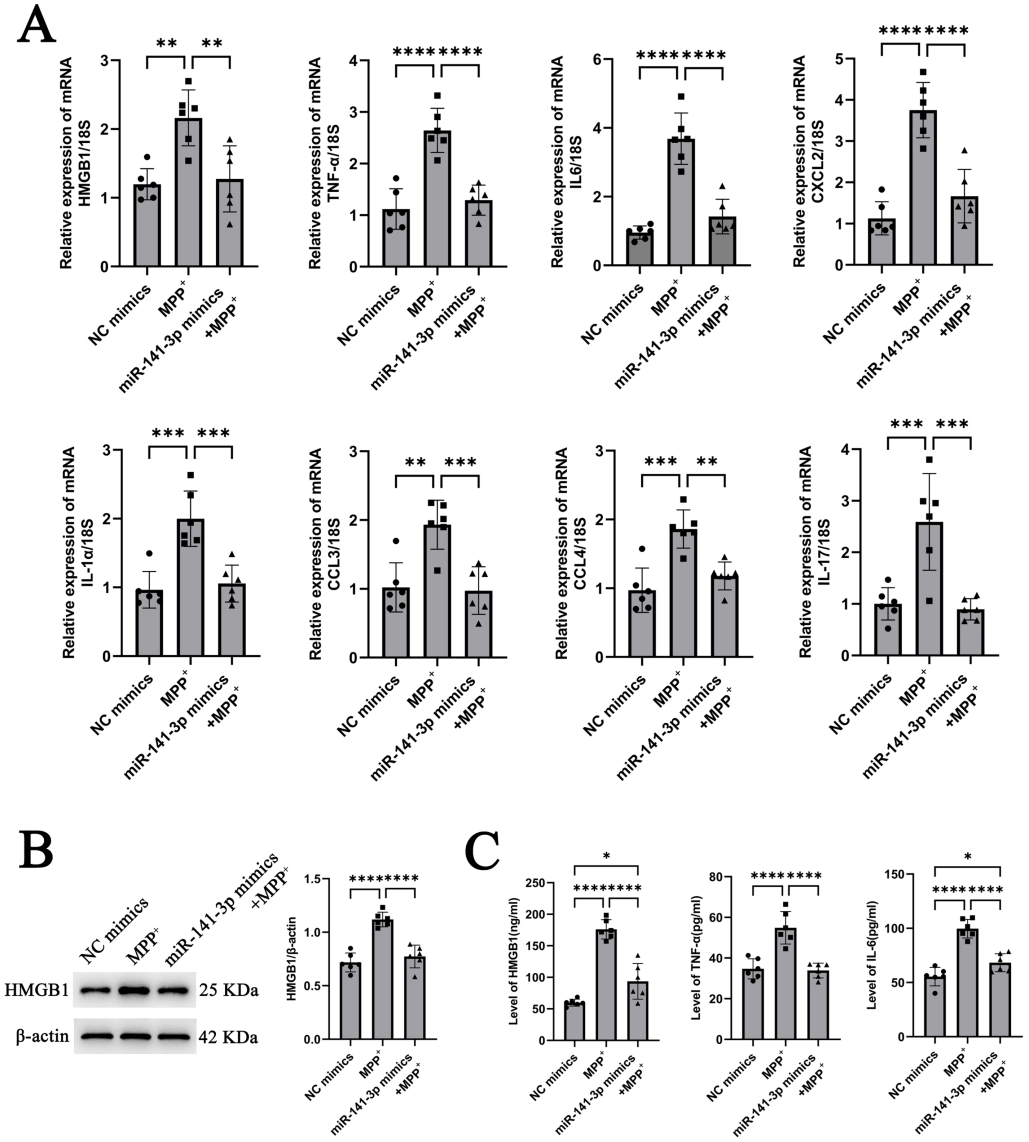
miR-141-3p mimic treatment reduced the expression of HMGB1 and pro-inflammatory cytokines in BV2 cells at 24 h after MPP^+^ treatment. **(A)** Histogram showed qRT-PCR assay to determine mRNA levels of HMGB1, IL-6, TNF-α, CXCL2, IL-1α, CCL3, CCL4, and IL-17 (*n* = 6 per group, **p < 0.01, ***p < 0.001, ****p < 0.0001, one-way ANOVA). **(B)** Western blotting assay showed the expression of HMGB1 (*n* = 6 per group, ****p < 0.0001, one-way ANOVA). **(C)** Histogram showed ELISA assay to determine the levels of HMGB1, IL-6 and TNF-α (*n* = 6 per group, *p < 0.05, ****p < 0.0001, one-way ANOVA).

To further assess the functional impact of miR-141-3p on HMGB1, *in vitro* experiments were conducted using miR-141-3p mimics. MPP^+^ stimulation was found to promote the HMGB1 mRNA expression in BV2 cells. However, upon transfection of miR-141-3p mimics into BV2 cells (miR-141-3p was overexpressed after transfection with miR-mimics as shown in Supplementary Figure S1), a significant reduction in HMGB1 mRNA expression was observed compared to the MPP^+^ group (Figure 2A). Additionally, the mRNA expressions of multiple pro-inflammatory factors, including IL-6, TNF-*α*, CXCL2, IL-1α, CCL3, CCL4, and IL-17, were significantly downregulated relative to the MPP^+^ group (Figure 2A). At the protein level, compared with the MPP^+^ group, the miR-141-3p mimics + MPP^+^ group exhibited a significant decrease in HMGB1 protein expression, with statistical significance (Figure 2B). ELISA quantification of HMGB1, TNF-α and IL-6 in the supernatant of BV2 cells revealed that the expression levels of these inflammatory factors were also significantly reduced in the miR-141-3p mimics + MPP^+^ group, compared to the MPP^+^ group (Figure 2C). These results indicate that miR-141-3p mimics can effectively reduce the expression and release of HMGB1, the release of pro-inflammatory factors from microglia at both the mRNA and protein levels.

### 3.3 miR-141-3p alleviates motor disorders in MPTP model

**Figure 3:**
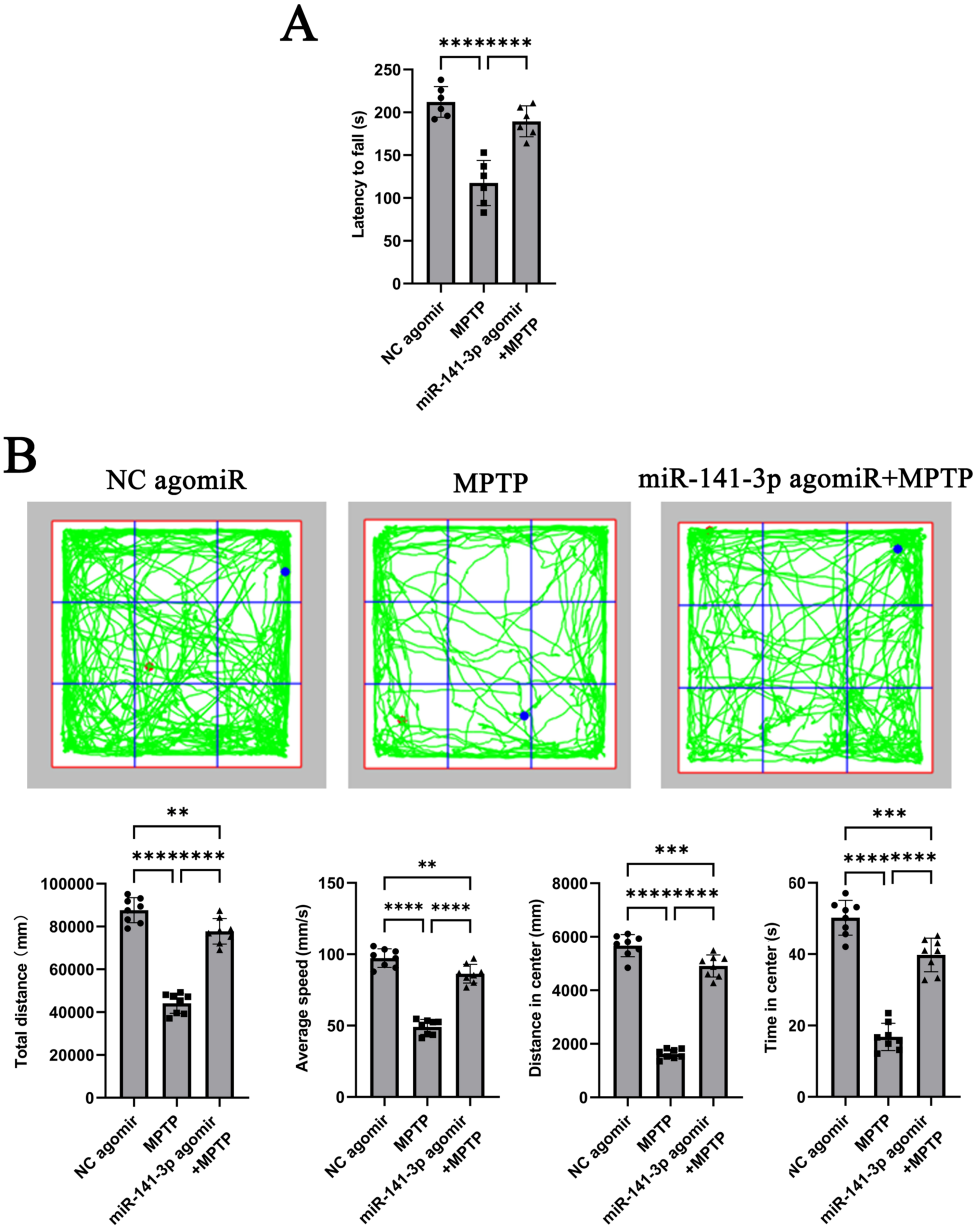
miR-141-3p agomir ameliorated motor deficits in the acute MPTP-induced mouse model of PD. **(A)** Forty-eight hours after intranigral injection of *miR*-141-3p agomir into the SNpc, mice received four intraperitoneal injections of MPTP within a single day. Rotarod test was performed 7 days post-MPTP administration. The histogram shows latency to fall from the rod (*n* = 8 per group; ****p < 0.0001, one-way ANOVA). **(B)** Open-field test was conducted 7 days after MPTP injection. The grid plot illustrates the movement trajectory of a representative mouse over a 15-min session. Histograms showed total distance traveled, average speed, distance moved, and time spent in the center zone (*n* = 8 per group, **p < 0.01, ***p < 0.001, ****p < 0.0001, one-way ANOVA).

To further validate the conclusion obtained from the *in vitro* experiments, stereotactic injections were performed on C57BL/6 J mice. The mice were then divided into three groups: NC agomir + 0.9% NaCl (referred to as NC agomiR), NC agomir + MPTP (referred to as MPTP), and miR-141-3p agomir + MPTP. Seven days later, behavioral tests were conducted on each group of mice. In the rotarod test, mice in the miR-141-3p agomir + MPTP group remained on the rotarod for a significantly longer period compared to the MPTP group. This finding suggests that miR-141-3p agomir enhances the balance and coordination abilities of MPTP model (Figure 3A). In the open field test, although the movement parameters did not fully return to normal levels, the total distance traveled and average speed of the miR-141-3p agomir + MPTP group were significantly higher than those of the MPTP group. Moreover, in the central area of the nine-grid, the movement distance and time of the miR-141-3p agomir + MPTP group were also significantly greater than those of the MPTP group. These data suggest that miR-141-3p agomir improves the spontaneous activity and exploration behavior of MPTP model (Figure 3B).

### 3.4 miR-141-3p alleviates pro-inflammatory factor expression in the SNpc region of MPTP model, reduces the number of reactive microglia, and increases the number of TH-positive neurons

**Figure 4:**
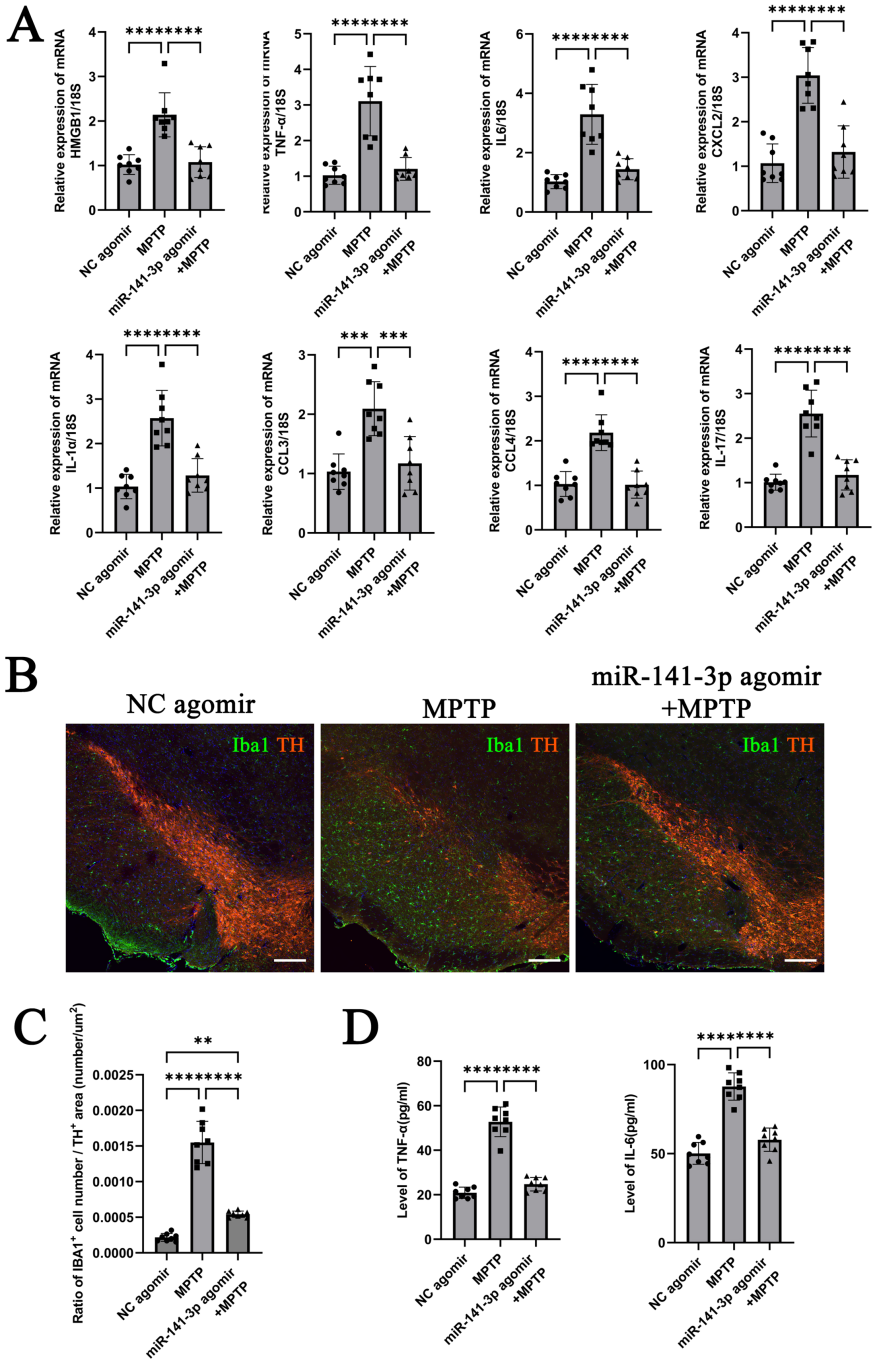
miR-141-3p agomir reduced the release of inflammatory factors, decreased microglial reaction, attenuated MPTP-induced dopaminergic neuronal damage in the midbrain SNpc. **(A)** Histogram showed qRT-PCR assay to determine mRNA levels of HMGB1, IL-6, TNF-α, CXCL2, IL-1α, CCL3, CCL4 and IL-17 in SNpc area (*n* = 8 per group, ***p < 0.001, ****p < 0.0001, one-way ANOVA). **(B)** The immunofluorescence technique showed the expression of Iba1 (green) and TH (red) in SNpc (Scale bar = 160 m). **(C)** Histogram showed the Iba1-positive cells number/TH-positive area ratio (*n* = 8 per group, **p < 0.01, ****p < 0.0001, one-way ANOVA). **(D)** Histogram showed ELISA assay to determine the levels of IL-6 and TNF-αin SNpc (*n* = 8 per group, ****p < 0.0001, one-way ANOVA).

After behavioral assessments, brain tissues from the lower half of the midbrain (including the SNpc region) were collected for analysis. Quantitative analysis of mRNA revealed that the miR-141-3p agomir + MPTP group had significantly reduced HMGB1 expression compared to the MPTP group. Additionally, the mRNA expression of IL-6, TNF-*α*, CXCL2, IL-1α, CCL3, CCL4 and IL-17 were significantly lower than those of the MPTP group (Figure 4A). Immunohistochemical fluorescence staining showed that compared to the NC agomiR group, the MPTP group exhibited extensive loss of TH-positive neurons in the SNpc region, accompanied by a significant increase in Iba1-positive microglial cell numbers, indicating a reactive state. In contrast, the number of TH-positive neurons in the miR-141-3p agomir + MPTP group was partially restored, and the number of Iba1-positive microglia decreased (Figure 4B, enlarged versions were shown in Supplementary Figure S2). The quantitative statistical analysis used the ratio of the number of Iba1-positive microglia to the area of TH-positive neurons. The results demonstrated a statistically significant difference between the MPTP group and the miR-141-3p agomir+MPTP group (Figure 4C). ELISA quantification demonstrated significantly reduced protein levels of pro-inflammatory cytokines IL-6 and TNF-α in the SNpc region of mice treated with miR-141-3p agomir+MPTP compared to the MPTP group (Figure 4D).

### 3.5 Inhibition of miR-141-3p reverses the effect of OMT on improving motor dysfunction in MPTP model

**Figure 5:**
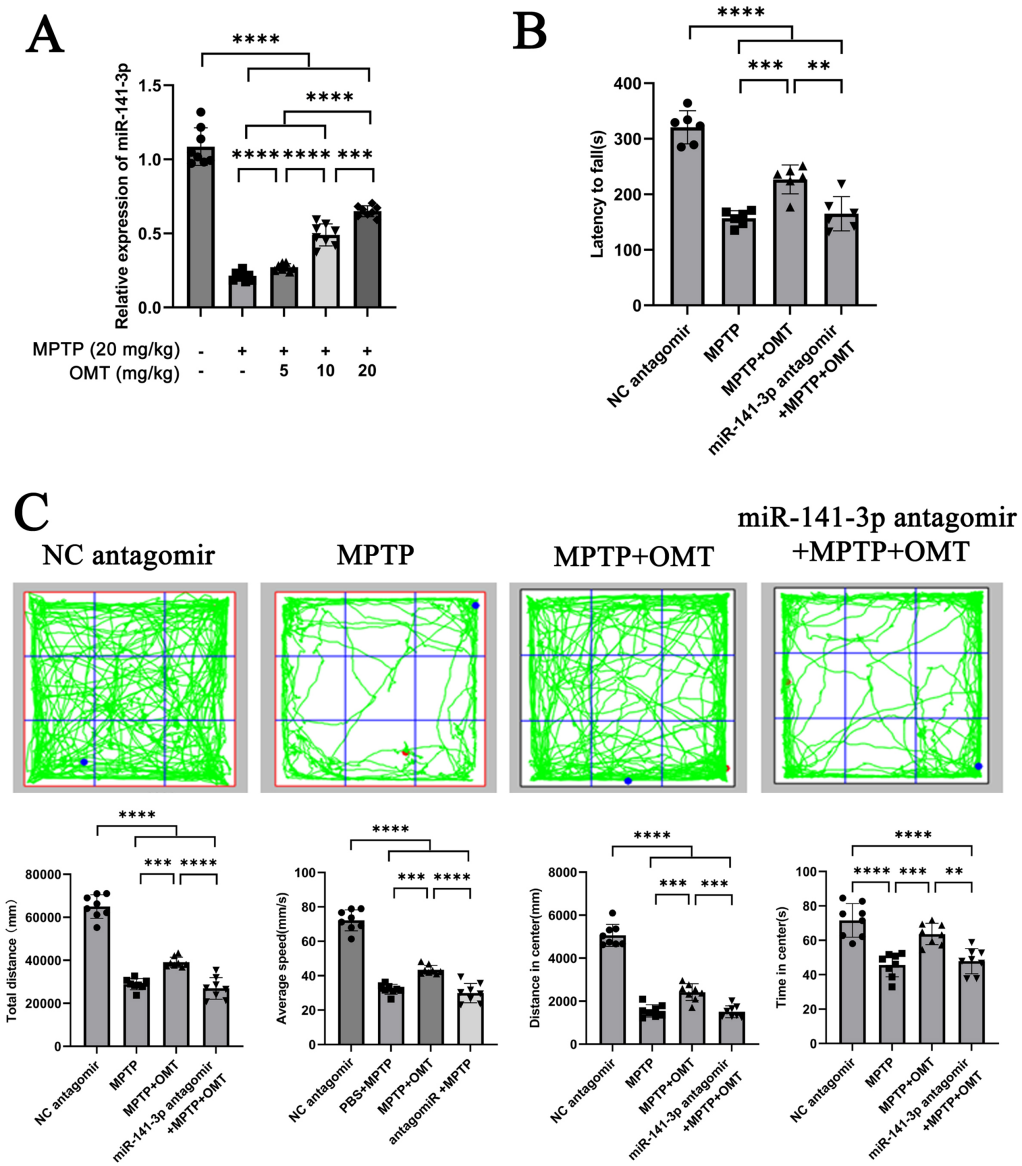
miR-141-3p antagomir reversed the beneficial effects of OMT on motor deficits in MPTP model. **(A)** Histogram showed qRT-PCR assay to determine the relationship between the expression level of miR-141-3p in SNpc and the dosage of OMT (*n* = 6 per group, ***p < 0.001, ****p < 0.0001, one-way ANOVA). **(B)** Forty-eight hours after the intranigral injection of miR-141-3p antagomir into the SNpc, mice received four intraperitoneal injections of MPTP within a single day, and subsequently treated with OMT (20 mg/kg, i.p.) for 7 consecutive days. Motor coordination was then assessed using the rotarod test. The histogram revealed the latency to fall from the rotating rod (*n* = 8 per group, **p < 0.01, ***p < 0.001, ****p < 0.0001, one-way ANOVA). **(C)** Open-field test was also performed. The grid plot illustrated the movement trajectory of a representative mouse over a 15-min session. Histograms showed total distance traveled, average speed, distance moved, and time spent in the center zone (*n* = 8 per group, **p < 0.01, ***p < 0.001, ****p < 0.0001, one-way ANOVA).

These data indicate that miR-141-3p regulates HMGB1 and modulates inflammatory and behavioral readouts in this MPTP paradigm. Building upon previous findings that OMT alleviates DA neuronal damage and microglia-mediated neuroinflammation (Gan et al., 2020), we investigated whether this effect occurs through miR-141-3p modulation. In MPTP-induced PD models, 7 days of intraperitoneal OMT treatment at varying doses increased miR-141-3p mRNA levels in the SNpc in a dose-dependent manner. (Figure 5A). Based on this result, we stereotaxically injected either NC antagomir or a miR-141-3p antagomir into the bilateral SNpc region. Ultimately, the experimental animals were divided into four groups: NC antagomir + 0.9% NaCl (referred to as NC antagomir), NC antagomir + MPTP (referred to as MPTP), NC antagomir + MPTP + OMT (referred to as MPTP + OMT), and miR-141-3p antagomir + MPTP + OMT. Behavioral assessments using the rotarod (Figure 5B) and open field tests (Figure 5C) showed that the miR-141-3p antagomir blocked the therapeutic benefits of OMT, impairing motor coordination, balance, and exploratory behavior.

### 3.6 Inhibition of miR-141-3p reverses the effects of OMT, exacerbating SNpc inflammation, microglial reaction, and TH-positive neuron loss

**Figure 6:**
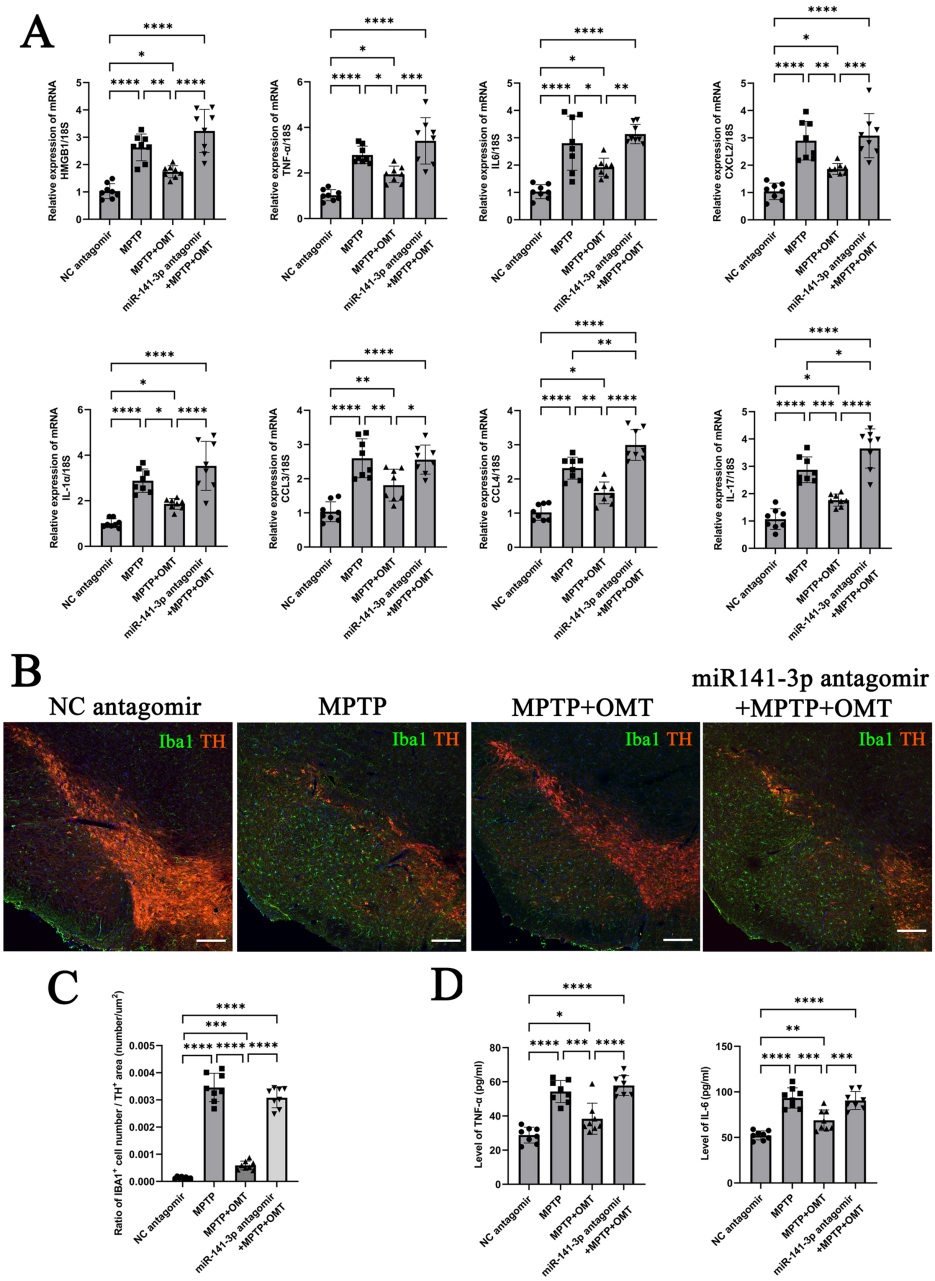
miR-141-3p antagomir reversed the neuroprotective effects of OMT in vivo, exacerbated the release of inflammatory factors, microglial reaction, and dopaminergic neuronal loss in the midbrain SNpc. **(A)** Histogram showed qRT-PCR assay to determine mRNA levels of HMGB1, IL-6, TNF-α, CXCL2, IL-1α, CCL3, CCL4 and IL-17 in SNpc area (*n* = 8 per group, *p < 0.05, **p < 0.01, ***p < 0.001, ****p < 0.0001, one-way ANOVA). **(B)** The immunofluorescence technique showed the expression of Iba1 (green) and TH (red) in SNpc (Scale bar = 160 m). **(C)** Histogram showed the Iba1-positive cells number/TH-positive area ratio (*n* = 8 per group, ***p < 0.001, ****p < 0.0001, one-way ANOVA). **(D)** Histogram showed ELISA assay to determine the levels of IL-6 and TNF-αin SNpc (*n* = 8 per group, *p < 0.05, **p < 0.01, ***p < 0.001, ****p < 0.0001, one-way ANOVA).

Compared with the MPTP + OMT group, the expression of HMGB1 in the miR-141-3p antagomir + MPTP + OMT group was significantly increased, consistent with the trend in the MPTP group. The mRNA expression of pro-inflammatory factors (IL-6, TNF-*α*, CXCL2, IL-1α, CCL3, CCL4, and IL-17) showed similar upregulation patterns (Figure 6A). Iba1 and TH double-labeling in the SNpc revealed that OMT treatment attenuated the MPTP-induced loss of TH-positive neurons and reduced the number of reactive microglia. Conversely, these protective effects were abolished by miR-141-3p antagomir co-treatment, which resulted in a pronounced loss of TH-positive neurons and a concomitant increase in reactive microglia. (Figure 6B, enlarged versions were shown in Supplementary Figure S3). The Iba1 number/TH area ratio also indicated that compared with the MPTP + OMT group, the ratio in the antagomir + MPTP + OMT group significantly increased, consistent with the trend in the MPTP group (Figure 6C). ELISA quantitative detection confirmed elevated IL-6 and TNF-α protein levels in antagomir + MPTP + OMT, compared with the MPTP + OMT group (Figure 6D).

### 3.7 Behavior after unilateral injections of miR-141-3p agomir or antagomir proves OMT can improve the behavioral disorders in MPTP model

In our research results, miR-141-3p agomir and miR-141-3p antagomir were injected bilaterally into the SNpc region. To evaluate the role of miR-141-3p, we performed unilateral injections of miR-141-3p agomir or antagomir into the left SNpc, followed by MPTP administration 48 h later. Mice in the miR-141-3p agomir + MPTP group exhibited ipsilateral rotation (leftward), indicating stronger neuroprotection in the injected (left) SNpc. In contrast, mice treated with miR-141-3p antagomir + MPTP + OMT exhibited contralateral rotation (rightward), indicating a loss of OMT-mediated protection in the injected hemisphere (Supplementary Video).

## 4 Discussion

As a fundamental innate immune response, inflammation initially protects against pathogens and facilitates tissue repair. Conversely, long-term continuous inflammatory stimulation, that is chronic inflammation, exacerbates tissue damage and is strongly implicated in the pathogenesis of multiple neurodegenerative diseases, including Alzheimer’s disease, Parkinson’s disease, and multiple sclerosis (McGeer et al., 1988; Raine, 1994; Banati et al., 1998). Microglia are resident immune cells of the CNS that maintain neural homeostasis. Upon transition to a reactive phenotype under pathological conditions, they secrete neurotoxic mediators—including TNF-*α*, IL-6, NO, PGE2, and superoxide—that damage neighboring cells and drive neurodegeneration (Kim and Joh, 2006).

We measured the expression of multiple pro-inflammatory factors *in vivo* and *in vitro* experiments. Upregulation of seven pro-inflammatory factors was presented to be suppressed by miR-141-3p. Among them, TNF-α is an important factor in regulating the inflammatory process and apoptotic cell death (Locksley et al., 2001). In postmortem brains from PD patients, TNF-α-producing glial cells are present, and TNF-α receptor immunoreactivity is evident on the soma and processes of most dopaminergic (DA) neurons (Boka et al., 1994; Mogi et al., 1994). Transcriptomic meta-analysis revealed that microglia responses to IL-6 is highly stimulus-specific, wide ranging and give rise to divergent phenotypes in neurodegenerative and neuroinflammatory disorders, such as Alzheimer’s disease, tauopathy, multiple sclerosis and lipopolysaccharide-induced endotoxemia (West et al., 2022). Elevated IL-6 levels are well-documented in the blood and brains of both human PD patients and animal models. DA neurons express IL-6 receptors, suggesting a direct pathway for IL-6-mediated toxicity. Accordingly, IL-6 receptor blockade using neutralizing antibodies protects against DA neuronal degeneration (Pons-Espinal et al., 2024). The findings indicate that TNF-*α* and IL-6 play significant roles in the pathological progression of Parkinson’s disease. CXCL2 has been demonstrated to be a pivotal driver of neutrophil migration to inflammatory sites in various disease models (Qin et al., 2017). CCL3 mainly participates in the pathological process of diseases such as AD by recruiting immune cells and driving neuroinflammation, while CCL4 exacerbates the pathological development of AD by aggravating neuroinflammatory responses and impairing the ability of glia cells to clear Aβ (Wang et al., 2024). IL-17 binding to its ubiquitously expressed receptor (IL-17R) activates downstream signaling, triggering the production of pro-inflammatory mediators and chemokines. This pathway is a key driver of autoimmune pathologies—including psoriasis, rheumatoid arthritis, and multiple sclerosis—by mediating inflammation and tissue injury (Storelli et al., 2019). Increased expression of IL-1α, CCL3, CCL4, and IL-17 has also been observed (Sznejder-Pachołek et al., 2017; Storelli et al., 2019; Chen et al., 2021; Leggio et al., 2022; Mohamed et al., 2023; Iba et al., 2024), but related research is still limited and not in-depth. The role of these pro-inflammatory factors in the pathological process of PD still needs further study.

HMGB1, an inflammatory amplifier released by glia and degenerating neurons, exacerbates neuroinflammation and secondary damage via the TLR4/NF-κB pathway (Takizawa et al., 2017; Wang et al., 2017; Wang et al., 2021). It promotes microglial reaction and the secretion of neurotoxic factors, including TNF-α, IL-1β, IL-6, and NO. Studies using LPS- or MPP^+^-induced neuron–glia co-cultures have shown that neutralization of HMGB1 with specific antibodies attenuates dopaminergic neuronal degeneration and injury (Gao et al., 2011). Similarly, in rat PD models, intravenous administration of anti-HMGB1 monoclonal antibodies (mAbs) can suppresses microglial reaction in the SNpc and reduces dopaminergic neuronal loss (Sasaki et al., 2016). These findings suggest that HMGB1 may be a promising therapeutic target that links neuroinflammatory responses to neurodegenerative processes in PD. However, the clinical application of heterologous anti-HMGB1 mAbs may be limited by potential side effects. Consequently, developing appropriate therapeutic strategies for anti-HMGB1 treatment remains an important research direction.

More than 70% of known microRNAs are enriched in the brain, where they play critical roles in neural development (Kosik, 2006). Among these, miR-150 levels in PD patients negatively correlate with proinflammatory cytokine expression. In microglia, LPS downregulates miR-150, whereas its overexpression attenuates neuroinflammation (Li et al., 2020). MicroRNA-155 is involved in LPS-mediated neuroinflammatory responses in mice (El-Sahar et al., 2021). Accumulating evidence indicates that multiple miRNAs are differentially expressed or dysregulated in PD and may contribute to disease pathogenesis by modulating downstream signaling pathways (Hernandez-Rapp et al., 2017; Saraiva et al., 2017; Juźwik et al., 2019). For instance, miR-141-3p directly targets TRAF5 and TRAF6, key regulators of NF-κB signaling, thereby inhibiting pathway activation and reducing the invasion, migration, and bone metastasis ability of prostate cancer cells (Huang et al., 2017; Luo et al., 2022). In T-cell acute lymphoblastic leukemia, miR-141-3p also suppresses NF-κB activation via TRAF5 (Zhou R. et al., 2019). Conversely, in certain contexts, miR-141-3p can be transferred to endothelial cells by small extracellular vesicles, activating the NF-κB pathway, which may be associated with angiogenesis in the tumor microenvironment (Masoumi-Dehghi et al., 2020). Previous studies have shown that miR-141-3p can down-regulate HMGB1 expression, alleviate inflammatory response in bacterial meningitis, and relieve chronic inflammatory pain caused by complete Freudner’s adjuvant (Shen et al., 2017; Fang et al., 2021). In this study, we initially identified an inverse correlation between miR-141-3p and HMGB1 expression in both BV2 microglia exposed to MPP^+^ and MPTP-treated mice. Subsequent TargetScan prediction and dual-luciferase reporter assays confirmed that miR-141-3p specifically targets HMGB1.

Numerous studies have reported that various Chinese herbal medicines or formulations can modulate microRNA expression. For instance, Baicalin downregulates miR-338-3p (Duan et al., 2019), Matrine suppresses miR-182-5p (Fu et al., 2020), and the combination of Hedyotis diffusa and Scutellaria barbata reduces miR-155 levels (Pan et al., 2016), and Triptolide lowers miR-21 (Sun et al., 2022). In contrast, Artesunate upregulates miR-16 (Zuo et al., 2014), and Pien Tze Huang enhances the expression of several key microRNAs, particularly miR-16 (Qi et al., 2017). Ethanol extract of Spica Prunellae elevates miR-34a (Fang et al., 2017), and luteolin promotes miR-34a expression (Wu et al., 2015). Additionally, Zhoushi Qi Ling decoction induces apoptosis in prostate cancer cells by upregulating miR-143 expression (Cao et al., 2021). OMT, an effective component of the traditional Chinese medicine *Sophora flavescens* with the molecular formula C₁₅H₂₄N₂O₂ (MW: 264.36), exhibits broad pharmacological properties including anti-tumor, anti-inflammatory, antiviral, anti-fibrotic, and immunomodulatory activities (Gao et al., 2018; Liang et al., 2018). Previous studies have shown that OMT modulates microRNA expression. It upregulates miR-520 to suppress VEGF and inhibit lung cancer cell proliferation and migration (Zhou W. et al., 2019), and downregulates miR-27a-3p to impede hemangioma stem cell proliferation, migration and induce their apoptosis via the PPAR-*γ* pathway (Dai et al., 2023), and downregulates miR-539-5p, altering the intestinal microbiota through the miR-539-5p/OGN/Runx2 pathway, reducing LPS release, and promoting osteoblast growth and differentiation, thereby improving diabetic osteoporosis (Zhang et al., 2025). In our study, OMT dose-dependently increased miR-141-3p expression in the SNpc of MPTP model. This upregulation targeted HMGB1, attenuating microglia-mediated neuroinflammation and dopaminergic neuronal damage via the HMGB1/TLR4/NF-κB pathway. Of course, this work focuses on a mouse model. In the future, we look forward to translating this finding into clinical applications to develop a practical and effective therapeutic strategy for PD.

## 5 Conclusion

miR-141-3p targets HMGB1 to inhibit microglial reaction and mitigate neuroinflammation both *in vivo* and vitro experiments, reduce TH-positive neuronal loss in the MPTP model. OMT increases miR-141-3p in the MPTP model, alongside reduced HMGB1 and inflammatory readouts, and these effects are diminished by miR-141-3p inhibition.

## Data Availability

The datasets presented in this study can be found in online repositories. The names of the repository/repositories and accession number(s) can be found in the article/Supplementary material.
