# Supplementary material for: Oxymatrine-associated protection in an MPTP mouse model is accompanied by increased miR-141-3p and reduced HMGB1
Source: Front Mol Neurosci. 2026 Feb 5;19:1731850. doi: 10.3389/fnmol.2026.1731850 (PMC12916691; doi:10.3389/fnmol.2026.1731850)
Supplement: Supplementary file 1 [file Table_1.DOCX]

**Table 1**. The primer sequences

|  | Type | Sequence |
| --- | --- | --- |
| Mus-miR-141-3p | forward | GCCGAGTAACACTGTCTGGT |
|  | reverse | CTCAACTGGTGTCGTGGAGT |
| Mus-HMGB1 | forward | GCGAACACGGCGTGCTCTAAG |
|  | reverse | GGGTGCTTCTTCTTGTGCTCCTC |
| Mus-18S | forward | CAGACCACCCGAGATTGAGCA |
|  | reverse | TTGGTTGAGGGAATCATTCAT |
| Mus-IL-1α | forward | AGTAGCGACGGGCGGTGTG |
|  | reverse | TCCATAACCCATGATCTGGAA |
| Mus-IL-6 | forward | AGAGGATACCACTCCCAACA |
|  | reverse | CAGTTTGGTAGCATCCATCA |
| Mus-IL-17 | forward | GGACTGTGATGGTCAACCTGA |
|  | reverse | TCATGTGGTAGTCCACGTTCC |
| Mus-TNF-α | forward | GAACTGGCAGAAGAGGCACT |
|  | reverse | AGGGTCTGGGCCATAGAACT |
| Mus-CCL3 | forward | TTCTGCTGACAAGCTCACCCTC |
|  | reverse | GAGGAACGTGTCCTGAAGTCTTTC |
| Mus-CCL4 | forward | CATGAAGCTCTGCGTGTCTG |
|  | reverse | GAAACAGCAGGAAGTGGGAG |
| Mus-CXCL2 | forward | CCAACCACCAGGCTACAGG |
|  | reverse | GCGTCACACTCAAGCTCTG |
