# Supplementary material for: Oxymatrine-associated protection in an MPTP mouse model is accompanied by increased miR-141-3p and reduced HMGB1
Source: Front Mol Neurosci. 2026 Feb 5;19:1731850. doi: 10.3389/fnmol.2026.1731850 (PMC12916691; doi:10.3389/fnmol.2026.1731850)

1. **pMIR-REPORT vector system**

1. **Align the 3'UTR sequence of the HMGB1 gene on the BLAST website. The exact mutated nucleotides are highlighted by a red box. Query : WT , Sbjct****: MUT.**


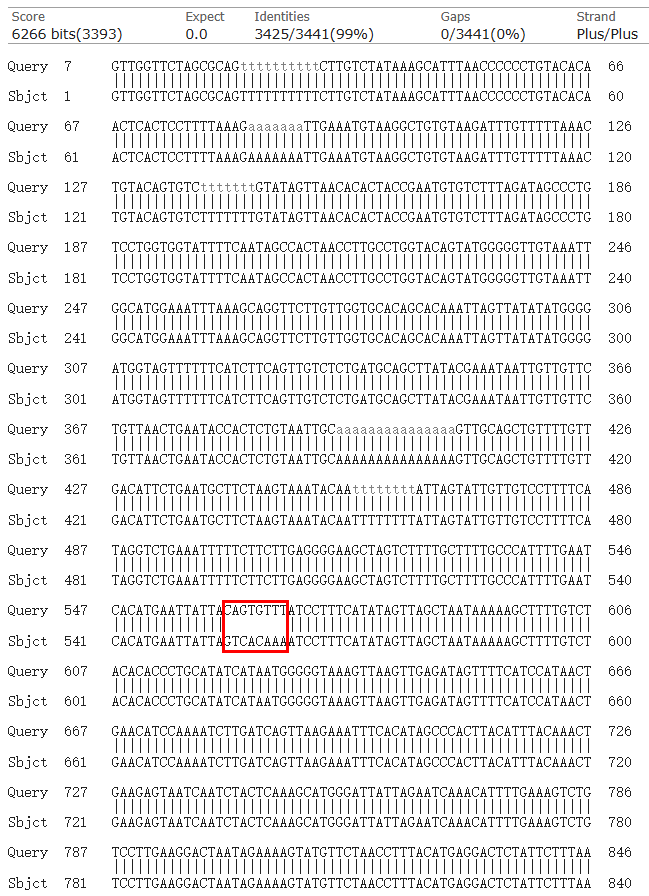


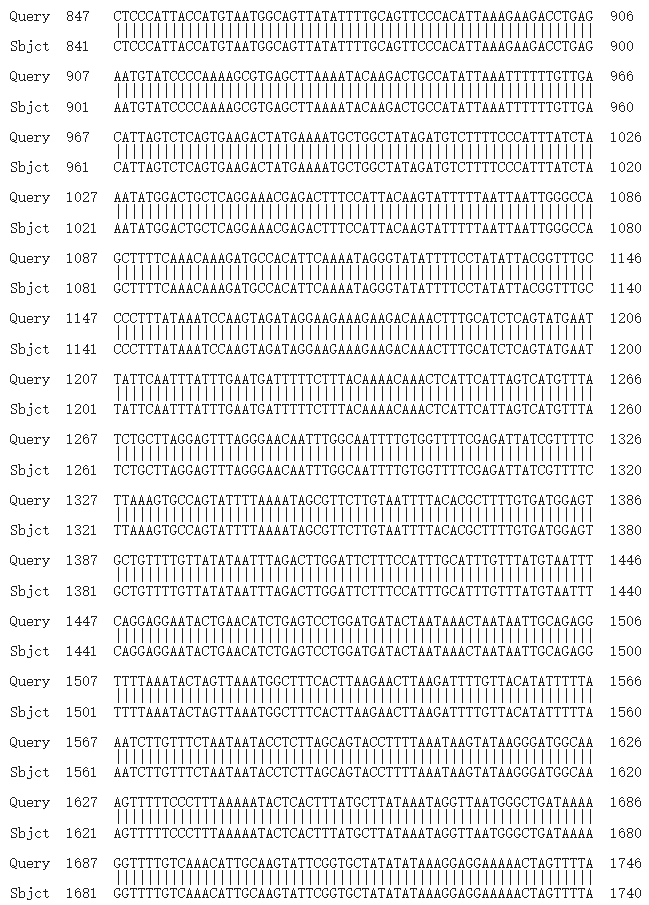


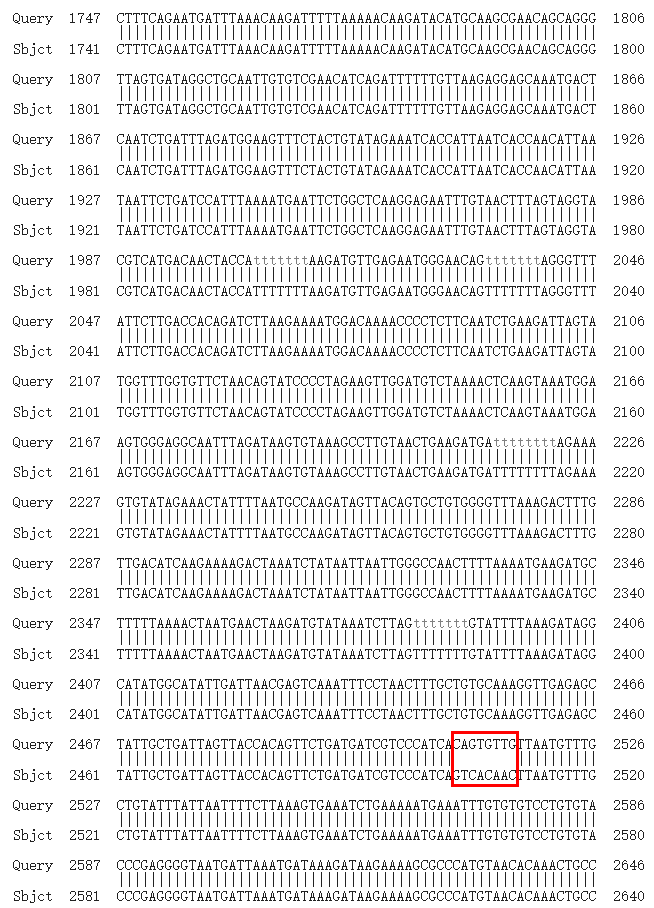


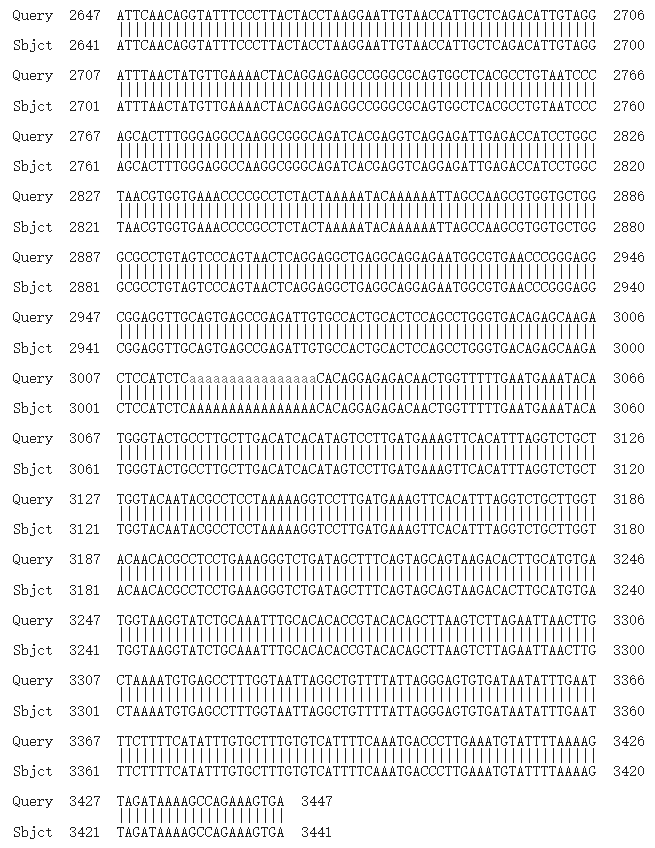

Supplement: Supplementary file 2 [file Table_2.DOCX]
