# Supplementary figures and images for: Oxymatrine-associated protection in an MPTP mouse model is accompanied by increased miR-141-3p and reduced HMGB1
Source: Front Mol Neurosci. 2026 Feb 5;19:1731850. doi: 10.3389/fnmol.2026.1731850 (PMC12916691; doi:10.3389/fnmol.2026.1731850)

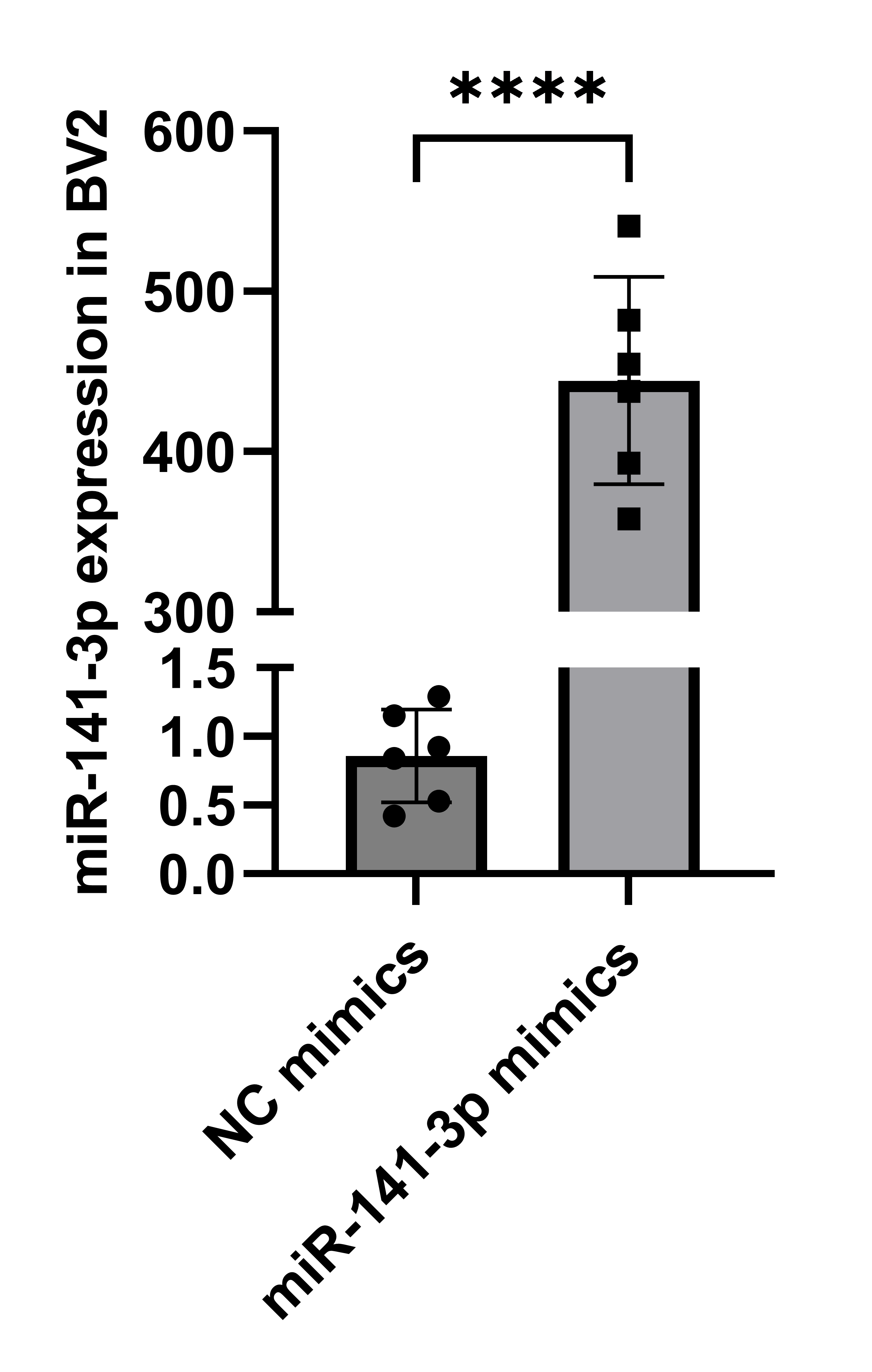

Supplement: Supplementary file 4 [file Image_1.TIF]

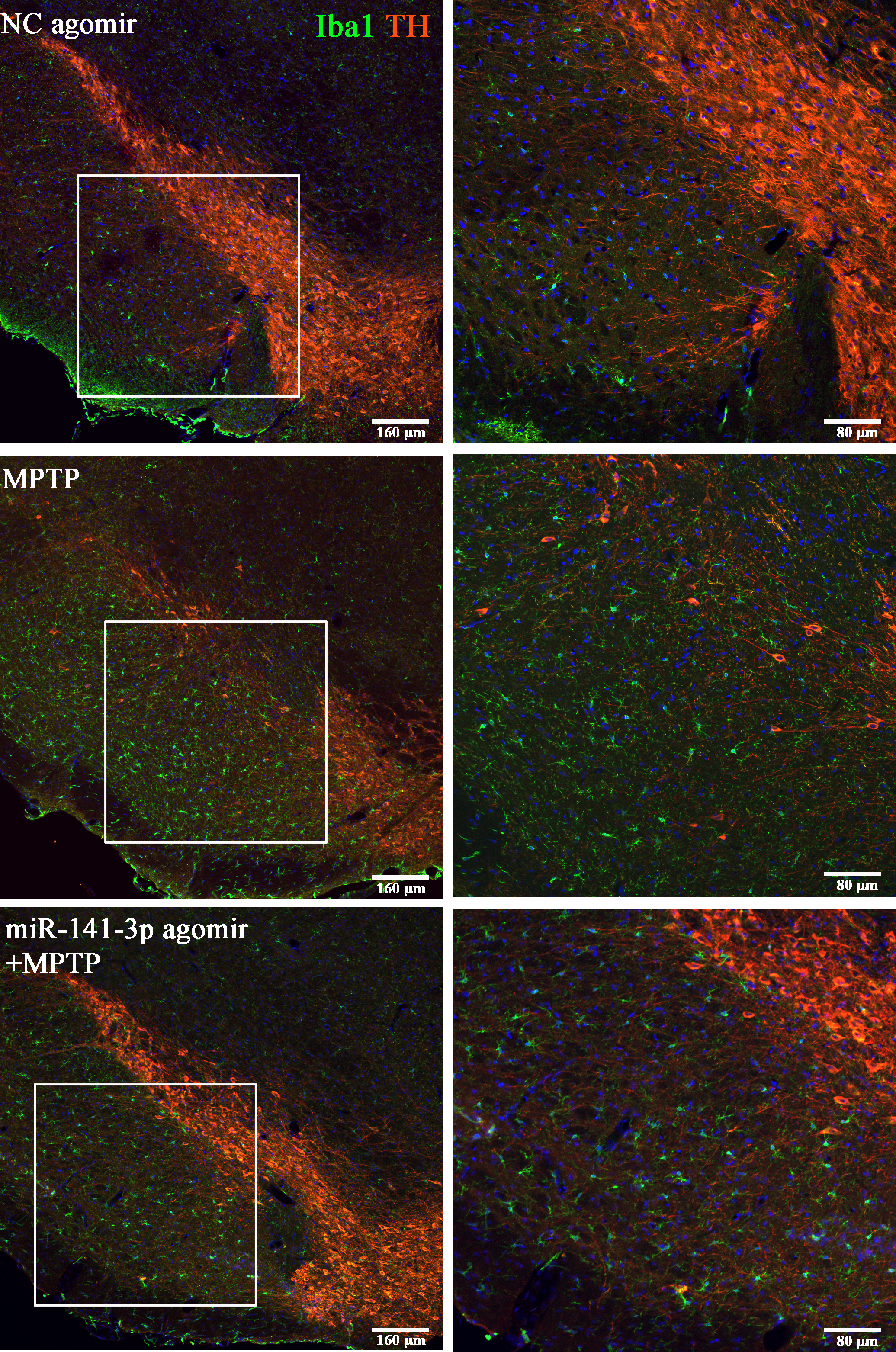

Supplement: Supplementary file 5 [file Image_2.JPEG]

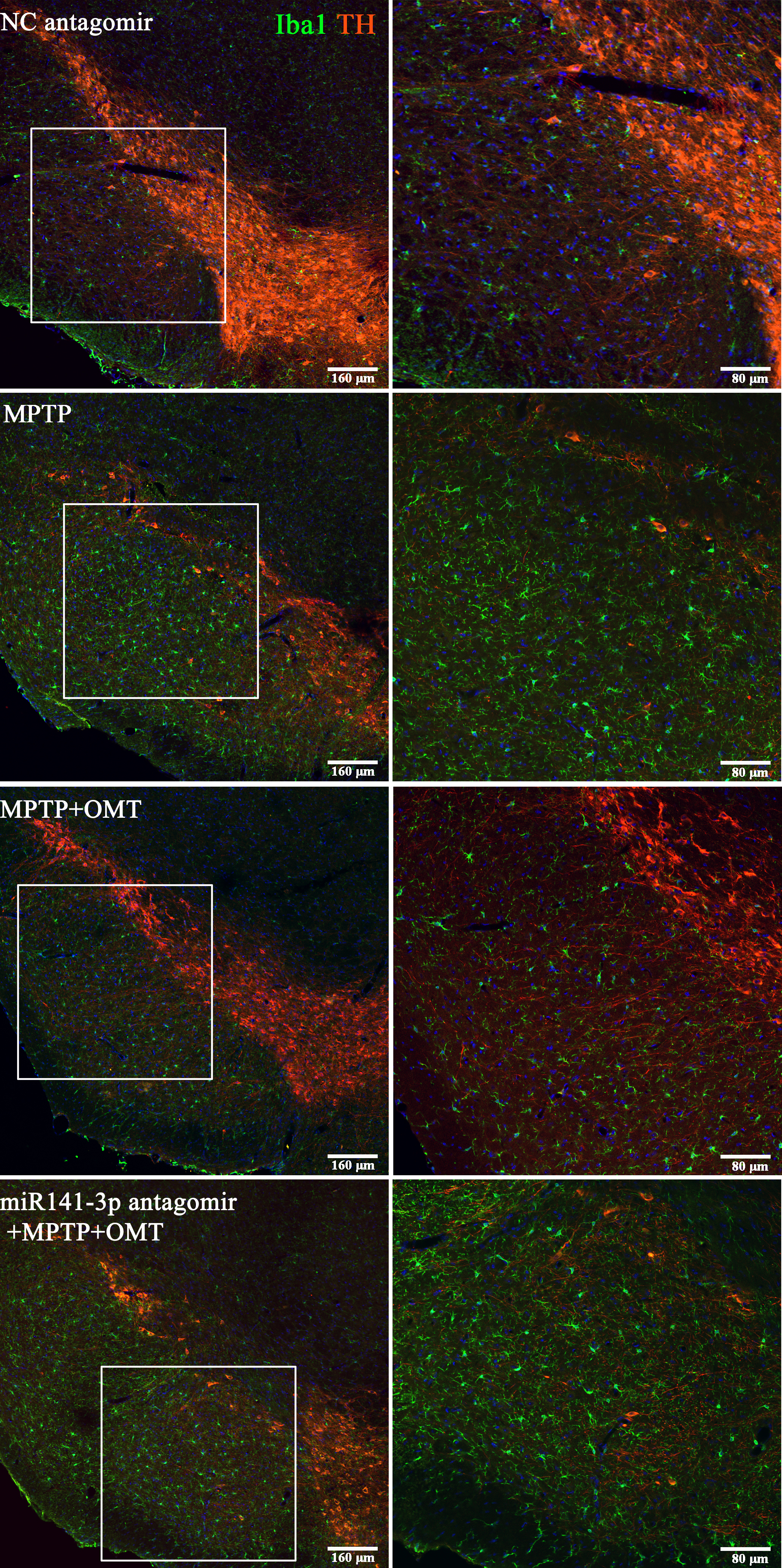

Supplement: Supplementary file 6 [file Image_3.JPEG]
